# Newcastle disease virus acquires phosphatidylserine through the budding process to enhance infectivity

**DOI:** 10.1080/21505594.2025.2580150

**Published:** 2025-10-28

**Authors:** Yuechi Hou, Lei Tan, Ping Zhang, Dandan Zhang, Libin Chen, Xusheng Qiu, Yingjie Sun, Cuiping Song, Ying Liao, Tao Ren, Chan Ding

**Affiliations:** a School of Agriculture and Biology, Shanghai Jiao Tong University, Shanghai Key Laboratory of Veterinary Biotechnology, Key Laboratory of Urban Agriculture (South), Ministry of Agri culture, Shanghai, China; b Department of Avian Diseases, Shanghai Veterinary Research Institute, Chinese Academy of Agricultural Sciences, Shanghai 200241, China; c College of Veterinary Medicine, South China Agricultural University, Guangzhou, China; d Jiangsu Co innovation Center for Prevention and Control of Important Animal Infectious Diseases and Zoonoses, Yangzhou 225009, PR China

**Keywords:** NDV, phosphatidylserine, scramblase, TMEM16F, apoptosis mimicry

## Abstract

Newcastle disease, a highly contagious avian illness caused by the Newcastle disease virus (NDV), inflicts substantial economic losses upon the global poultry industry. While NDV is known to enter host cells via multiple pathways, critical aspects of its infection and pathogenic mechanisms, particularly the role of host lipids, remain incompletely understood. Here, we demonstrate that NDV infection strategically manipulates host phosphatidylserine (PS) metabolism to enhance its replication cycle. We found that the NDV hemagglutinin-neuraminidase (HN) protein triggers an elevation in intracellular Ca^2+^ levels, which in turn activates the host phospholipid scramblase TMEM16F. This activation leads to the externalization of PS to the outer leaflet of the plasma membrane. Consequently, NDV virions budding from these PS-rich membrane domains acquire a PS-enriched envelope. Mass spectrometry analysis confirmed high PS abundance on the viral surface. These PS-decorated progeny virions then engage host cell PS receptors, specifically the receptor tyrosine kinase TYRO3 and T-cell immunoglobulin and mucin domain-containing receptor 4 (TIM-4), to facilitate enhanced viral adsorption and entry. This process, known as “apoptotic mimicry,” represents a novel, parallel entry pathway for NDV. These findings provide new mechanistic insights into NDV-host interactions and identify the PS scrambling and recognition axis as a potential therapeutic target for developing novel anti-NDV strategies.

## GRAPHICAL ABSTRACT

**Figure:**
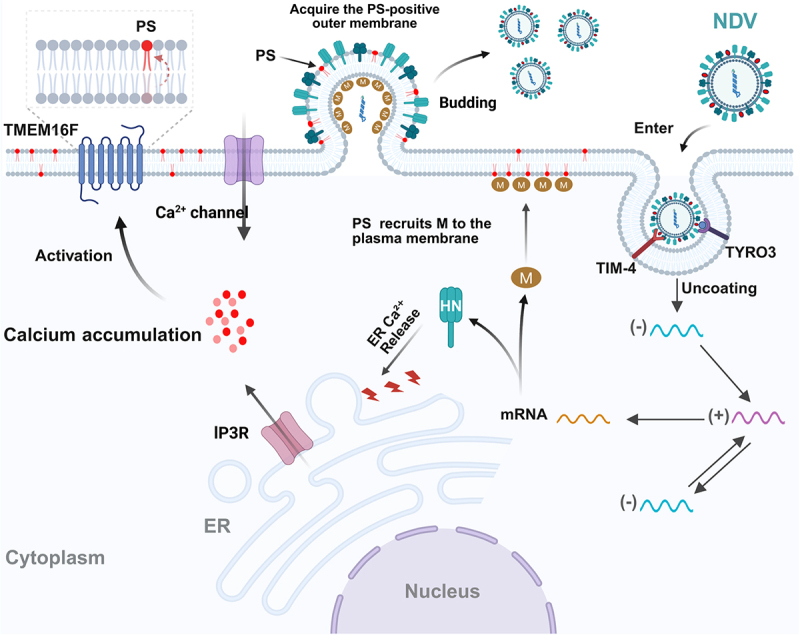

## Introduction

Newcastle disease virus (NDV), a member of the *Avulavirus* genus within the *Paramyxoviridae* family, is the causative agent of Newcastle disease, a devastating and highly contagious infection in poultry that results in significant global economic losses [1]. The economic impact of Newcastle disease is twofold, stemming from direct poultry mortality and from trade restrictions imposed on affected regions [2]. As an enveloped, negative-sense, single-stranded RNA virus [3], the initial step of NDV infection is mediated by the attachment of its hemagglutinin-neuraminidase (HN) glycoprotein to sialic acid-containing receptors on the host cell surface, followed by fusion of the viral and cellular membranes [3,4]. While NDV can enter cells through direct plasma membrane fusion or various endocytic pathways depending on the cell type [5]. The full repertoire of its invasion strategies remains to be elucidated [6].

Our previous study established that phospholipid metabolism critically influences NDV infection in host cells [7]. Phosphatidylserine (PS), a key structural phospholipid predominantly localized to the membrane’s inner leaflet, exhibits a negative charge [8]. Synthesized by PS synthases 1 and 2 (PSS1/2) through serine exchange reactions with phosphatidylcholine and phosphatidylethanolamine, respectively [9,10]. PS becomes externally exposed during apoptosis and platelet activation – serving as a critical signaling molecule [11]. In apoptotic cells specifically, this externalized PS acts as a clearance signal for phagocytosis by the monocyte-macrophage system [12]. Conversely, platelet surface PS exposure confers a negatively charged procoagulant surface for thrombin generation [13]. The phospholipid scramblase TMEM16F – primarily localized to the plasma membrane – mediates Ca^2 +^ -activated PS externalization from the inner to the outer membrane leaflet [14–16]. Elevated intracellular Ca^2 +^ stabilizes TMEM16F as a homodimer, triggering PS exposure [17,18]. However, how NDV activates TMEM16F in infected cells remains unknown. Critically, viral membrane composition mirrors that of the host cell membrane during assembly and budding [19]. Given PS’s essential role in membrane biology, we hypothesize that NDV exploits PS to facilitate viral assembly and budding.

Recent research shows some viruses have abundant PS around their envelopes, mimicking apoptotic fragments to exploit the apoptotic clearance mechanism. This “apoptotic mimicry” strategy, where PS decoration induces “eat me” signal uptake and evades immune response, enables viruses to infect diverse cell types without needing specific receptor-binding proteins [17,18,20,21]. Viruses that mimic apoptosis target a range of PS receptors, such as Tyro3-Axl-Mer (TAM) receptors (AXL, MERTK, and TYRO3), the T-cell immunoglobulin mucin receptor (TIM) family (TIM-1/3/4), CD300a, and MFG-E8, thereby facilitating viral entry into host cells [20]. A growing body of evidence indicates that enveloped viruses have evolved sophisticated strategies to exploit host cell lipids to facilitate their life cycle. For instance, the African swine fever virus (ASFV) envelope binds AXL to facilitate entry into porcine alveolar macrophages (PAMs) [22]. Furthermore, apoptotic body-associated ASFV virions are phagocytosed by neighboring PAMs via interactions between PS and specific receptors TIM-4/MFG-E8 [23]. Ebola virus (EBOV) similarly exploits TYRO3, AXL, and TIM-1 to enhance cellular invasion [24–26]. Notably, however, our prior study demonstrated that chicken MERTK negatively regulates NDV infection and replication in DF1 cells contrasting with ASFV/EBOV mechanisms [27]. We also found that NDV infection reprograms host phospholipid metabolism in chickens, utilizing cellular lipids to form new viral envelopes [7].

In this study, we investigated the molecular mechanisms by which NDV exploits host PS to facilitate its replication. We hypothesized that NDV not only requires host PS for the assembly and budding of new virions but also actively induces PS externalization to equip its progeny with a PS-rich envelope, thereby enabling entry via apoptotic mimicry. Our findings reveal that the NDV HN protein activates TMEM16F to promote PS exposure, leading to the production of PS-rich virions. We further identify TYRO3 and TIM-4 as key PS receptors that mediate the entry of these virions, thus uncovering a novel, parallel invasion pathway for NDV.

## Materials and methods

### Cells and viral strains

A549, HEK-293T and BHK-21 cells were obtained from the American Type Culture Collection (ATCC). HEK-293T and BHK-21 cells were cultured in Dulbecco’s modified Eagle’s medium (DMEM) supplemented with 10% fetal bovine serum (FBS) (Gibco, Franklin Lakes, NJ, USA). A549 cells were cultured in Roswell Park Memorial Institute-1640 (RPMI-1640) medium (Gibco, Franklin Lakes, NJ, USA). The NDV Herts/33 strain was obtained from China Institute of Veterinary Drug Control (Beijing, China). NDV GFP-Herts/33 and RFP- Herts/33 strains were created and stored in the laboratory. NDV titers were assessed in BHK-21 cells using the median tissue culture infective dose (TCID_50_).

### Reagents and antibodies

Recombinant anti-PTDSS1 (ab157222) and anti-PTDSS2 (ab183504) antibodies were purchased from Abcam (Cambridge, MA, USA). The anti-HA-tag (C29F4) rabbit antibody (3724), and anti-DYKDDDDK tag (D6W5B) rabbit antibody (14793) were acquired from Cell Signaling Technology (Danvers, MA, USA). The anti-ANO6 polyclonal antibody (PA5-88322), Alexa Fluor goat anti-rabbit-488 antibody (A11034), and Alexa Fluor goat anti-rabbit-594 antibody (A11037) were purchased from Invitrogen (Carlsbad, CA, USA). The Anti-β-actin antibody (AC006) and anti-GAPDH Rabbit antibody (A19056) were purchased from ABclonal (Wuhan, China). The anti-PS and anti-TIM4 antibodies were provided by Dr. Gao Peng (China Agricultural University). The anti-NDV-NP mAbs were developed and stored in the laboratory. The TMEM16F activity inhibitor Niclosamide (HY-B0497), intracellular calcium chelator BAPTA-AM (HY-100545), and Ionomycin (HY-13434) were purchased from MedChemExpress (Monmouth Junction, NJ, USA). Ca^2+^ indicator Fluo-4 AM (20551), transporter activity inhibitor Probenecid (20061), and Pluronic® F-127 (20053), used to increase the water solubility of Fluo-4 AM, were purchased from AAT Bioquest (Sunnyvale, CA, USA). The SYBR Green qPCR Mix (P2093b) was purchased from Dongsheng Biotechnology (Guangzhou, China). Phanta Max Super-Fidelity DNA Polymerase (P505) and the ClonExpress Ultra One Step Cloning Kit (C115) were purchased from Vazyme (Nanjing, China). Annexin V-FITC apoptosis detection kit (C1062L) was purchased from Biyuntian Biotechnology (Shanghai, China). CCK-solution (40203ES76) was purchased from YEASEN (Shanghai, China). Color pre-stained protein standard (BL712A) was purchased from Biosharp (Anhui, China)

### Viral infection of cells

The viral stock solution was diluted to the required multiplicity of infection (MOI) in serum-free medium. We infected the cells for 1 h at 37°C, then removed non-adsorbed virus particles. Subsequently, the cells were washed three times with PBS and culture was continued in maintenance medium at 37°C. Experiments on NDV were conducted in a biosafety-level-3 laboratory at South China Agricultural University.

### Plasmids

The open reading frames (ORFs) of PSS1 (NM_014754.3), PSS2(NM_001329544.2), TMEM16F (NM_001025356.3), TYRO3 (NM_006293.4), GAS6 (NM_000820.4), PROS1 (NM_001314077.2), and TIM-4 (NM_138379.3) were inserted into pCMV-HA (Clonetech) to construct plasmids with HA tags. The sequence of Bos taurus milk fat globule EGF and factor V/VIII domain containing (MFGE8) (NM_176610.1) was synthesized by Shanghai Sangon Biotech and subsequently inserted into the pEGFP-C1 vector to construct the fluorescent labeled plasmid Lact-C2-GFP. Plasmids encoding the NDV proteins NP, P, V, W, M, F, and HN were generously provided by Dr. Yuan Weifeng (South China Agricultural University, Guangzhou, China). All plasmid constructs were validated by Sanger sequencing. Table 1 details the primers used for plasmid construction. The P×459backbone was linearized with BbsI, and the annealed oligonucleotide duplexes encoding the sgRNAs were ligated downstream of the U6 promoter to generate CRISPR/Cas9 knockout constructs targeting PSS1 or PSS2. The guide sequences were PSS1-sgRNA: 5”-GATCAACGAGCAGCAAGTGG-3‘ and PSS2-sgRNA: 5’-GAGTCCGAGGTCTACGACGA-3.”

**Table 1.** The primer sequences related to plasmid construction.

Gene	Sequence (5′–3′)	TM	Length
PSS1	F: GGCCATGGAGGCCCGAATTCACatggcgtcctgcgtggg	62 ℃	1419 bp
	R: GATCCCCGCGGCCGCGGTACCtcatttctttccaacgccattggtgact		
PSS2	F: TGGCCATGGAGGCCCGAATTCACatgcggaggggcgagc	61 ℃	1509 bp
	R: GATCCCCGCGGCCGCGGTACCtcagtttggagttggtgctccct		
TMEM16F	F: TGGCCATGGAGGCCCGAATTCACatgaaaaagatgagcaggaatgt	55 ℃	2733 bp
	R: GATCCCCGCGGCCGCGGTACCttattctgattttggccgtaaattg		
TYRO3	F: TGGCCATGGAGGCCCGAATTCCAatggcgctgaggcgga	60 ℃	2673 bp
	R: GATCCCCGCGGCCGCGGTACCctaacagctactgtgtggcagtagc		
GAS6	F: TGGAGGCCCGAATTCCAatggccccttcgctctc	60 ℃	2037 bp
	R: GATCCCCGCGGCCGCGGTACCctaggctgcggcggg		
PROS1	F: TGGAGGCCCGAATTCCAatgagggtcctgggtggg	60 ℃	2127 bp
	R: GATCCCCGCGGCCGCGGTACCttaagaattctttgtctttttccaaactgatggac		
TIM-4	F: TGGCCATGGAGGCCCGAATTCCAatgtccaaagaacctctcattctct	58 ℃	1137 bp
	R: GATCCCCGCGGCCGCGGTACCttagagggtaaaaaggccgtct		

### RNA interference

siRNA oligonucleotides were purchased from GenePharma (Shanghai, China). The siRNA sequences are listed in Table 2. The efficacy of knockdown was confirmed by RT-qPCR or western blot analysis. The final concentration of the siRNA was 100 nM for single siRNA transfections.

**Table 2.** The siRNA sequences in this study.

siRNA	Sequence (5′–3′)	
si-TMEM16F	Sense	GGUGGCAAGAUCAUAAUGUTT
	Antisense	ACAUUAUGAUCUUGCCACCTT
si-AXL	Sense	GACUGUCUGGAUGGACUGUTT
	Antisense	ACAGUCCAUCCAGACAGUCTT
si-MERTK	Sense	AGAUGACAUGACUGUCUGUTT
	Antisense	ACAGACAGUCAUGUCAUCUTT
si-TYRO3	Sense	GCUUCGAAAGAGACGGAAATT
	Antisense	UUUCCGUCUCUUUCGAAGCAG
si-CD300a	Sense	CAGGGAAGAACUUCACUAUTT
	Antisense	AUAGUGAAGUUCUUCCCUGTT
si-MFG-E8	Sense	CCAGUCAUGAGUACCUGAATT
	Antisense	UUCAGGUACUCAUGACUGGTT
si-TIM-1	Sense	GACGGCCAAUACCACUAAATT
	Antisense	UUUAGUGGUAUUGGCCGUCTT
si-TIM-3	Sense	GAGCCUCCCUGAUAUAAAUTT
	Antisense	AUUUAUAUCAGGGAGGCUCTT
si-TIM-4	Sense	GCUGGUUCAACGAUGUAAATT
	Antisense	UUUACAUCGUUGAACCAGCTT

### Transfection of siRNA or plasmids

Expression plasmids or siRNAs were transfected into cells using Lipofectamine 2000 (Thermo Fisher Scientific) following the manufacturer’s instructions. Six hours post-transfection, the medium containing the transfection complex was replaced with fresh medium and the cells were cultured at 37°C with 5% CO_2_.

### Cell viability assay

Cell viability following drug treatment was evaluated using the CCK-8 assay. Cells were plated in 96-well plates and exposed to varying concentrations of Fendline, BAPTA-AM, Ionomycin, or niclosamide for 24 h. Subsequently, CCK-8 solution (10 μL) was added to each well, followed by a 1 h incubation period in the incubator. Then, absorbance at 450 nm was quantified using a microplate reader. Cell viability was calculated using the following formula: cell viability = [A (drug added) – A (blank)]/[A (0 drug added) – A (blank)] × 100%.

### SDS-PAGE and western blot analysis

Total protein was extracted from the cells using RIPA lysis buffer (Beyotime) and protease and phosphatase inhibitors (Beyotime) on ice. Subsequently, 1× SDS loading buffer (Beyotime) was added, and the mixture was boiled for denaturation for 15 min. Denatured samples were separated by SDS-PAGE and transferred onto nitrocellulose membranes (Whatman, Little Chalfont, UK). The membrane was then blocked in 5% skim milk for 1 h and incubated with the target antibody at 4°C. The membrane was then washed three times with Tris-buffered saline containing Tween 20 (TBST) for 10 min each. The membrane was then subjected to incubation with secondary antibody for 2 h at room temperature, followed by three washes with TBST for 10 min each. Protein bands were visualized using a Tanon 4600 chemiluminescence imaging system (Bio Tanon, Shanghai, China) and quantified using ImageJ software (National Institutes of Health, Bethesda, MD, USA).

### Quantitative real-time polymerase chain reaction

Total RNA was extracted using the TRIzol reagent (Invitrogen, Carlsbad, CA, USA) following the manufacturer’s instructions. Reverse transcription complementary DNA was synthesized using a reverse transcription kit with gDNA Eraser (11120ES60, YEASEN) according to the manufacturer’s protocol. SYBR Green qPCR Mix (P2093b, Dongsheng Biotech) was utilized for RT-qPCR, and quantification was performed using the ΔΔCt method. The primer sequences are listed in Table 3.

**Table 3.** The qPCR primer sequences in this study.

Gene	Sequence (5′–3′)		TM
β-actin	Forward	TGTGGCCGAGGACTTTGATT	60 ℃
	Reverse	CCTGTGTGGACTTGGGAGAG	
NDV NP	Forward	CAACAATAGGAGTGGAGTGTCTGA	60 ℃
	Reverse	CAGGGTATCGGTGATGTCTTCT	
PSS1	Forward	AGACCTACTCGGAGTGTGAAGATGG	60 ℃
	Reverse	CCTGGAAGAATGGCTTTCGTTGTTG	
PSS2	Forward	CTCACCCTGTCCCTGCCCTTC	60 ℃
	Reverse	TCATCCTTGTTCTGCCACTTCTGC	
TMEM16F	Forward	CAAGCCCGACCAGAATACGAAGC	60 ℃
	Reverse	CAGCACTGGCACAGAGGGTTATC	
AXL	Forward	GCTGGAGGTGGCTTGGACTC	60 ℃
	Reverse	ACGGATGCTTGCGAGGTGAG	
MERTK	Forward	GAGATGGCGGTCTTCAGTTGTG	60 ℃
	Reverse	GTGCTGTTACGGATGCTGACTTC	
TYRO3	Forward	CTGTGGTCCTTGGTGTGCTAAC	60 ℃
	Reverse	AAGTGAACGGCTGGCTCTCC	
CD300a	Forward	AGGTTGAGGTGTCCGTGTTCC	60 ℃
	Reverse	AGTTGTGATTGTTGAGGTCTTGGC	
MFG-E8	Forward	AACCTGCTGCGGAGGATGTG	60 ℃
	Reverse	AATTCGTGTCCATTAAGGCTGTAGG	
TIM-1	Forward	TTCCAACGACAACGAGCATTCC	60 ℃
	Reverse	GCTGAGGTGAAGATGGTGAAGTG	
TIM-3	Forward	CAGAGCGGAGGTCGGTCAG	60 ℃
	Reverse	TGCCACATTCAAACACAGGACAG	
TIM-4	Forward	CATAGAAGTGCCTGGCTGGTTC	60 ℃
	Reverse	GTGTGGTGGTGGTTGCTGTTC	

### Co-immunoprecipitation

HEK-293T cells were seeded in 6-cm dishes and grown to 70 % confluence before co-transfection with plasmids encoding HA-tagged HN and FLAG-tagged TMEM16F. Twenty-four hours later, cells were lysed on ice for 30 min with RIPA buffer. Lysates were incubated overnight at 4°C with anti-FLAG magnetic beads (Bimake, Houston, TX, USA). After three washes with PBST, bound proteins were eluted by boiling in 1× SDS loading buffer and analyzed by western blotting.

### Immunofluorescence assay

The cell culture medium was aspirated, and the cells were washed three times with PBS, fixed with 4% neutral formaldehyde for 20 min, and permeabilized with 0.5% Triton X-100 in TBST solution for another 20 min. Subsequently, we blocked the cells with 5% bovine serum albumin for 1 h at room temperature, followed by incubation with primary antibodies at 37°C for 2 h. After washing, the cells were incubated with fluorescent secondary antibodies at 37°C for another 2 h. The cells were then washed and stained with DAPI for 10 min, after which the excess DAPI was removed. Finally, the cells were observed using an LSM880 confocal microscope (Carl Zeiss, Jena, Germany), and images were analyzed using ImageJ software.

### PS detection

Cell surface PS was quantified using FCM. The cell culture medium was aspirated into a suitable centrifuge tube, adherent cells were washed three times with PBS, and an appropriate amount of EDTA-free trypsin cell digestion solution was added for cell digestion. The cells were lifted or dislodged with trypsin. Subsequently, the previously collected cell culture medium was added, and the cells were gently dislodged, transferred to a centrifuge tube, and centrifuged at 1,000 g for 5 min. The supernatant was discarded, 500 μL of PBS was added, and the cells were gently resuspended. The suspension cells were centrifuged again at 1,000 g for 5 min, the supernatant was discarded, and 195 μL of Annexin V-FITC conjugate solution was added to gently resuspend the cells. Following this, 5 μL of Annexin V-FITC was added and gently mixed. The cells were then incubated at room temperature in the dark for 10–20 min before being placed in an ice bath for FCM. Total cellular PS levels were quantified following plasma-membrane permeabilization with 0.1% Triton X-100.

Surface proteins of the virus particles were analyzed by dot blotting. Five microliters of purified virions obtained by sucrose gradient centrifugation were applied to a nitrocellulose membrane, with PBS serving as a control. The membrane was then dried at 37°C and the spotting process was repeated at the original position. The membranes were then blocked with 5% bovine serum albumin for 1 h at room temperature. The membrane was incubated overnight at 4°C with anti-PS and anti-NP antibodies. After three washes with TBST, the membrane was incubated with the secondary antibody for 2 h at room temperature, followed by three additional washes with TBST. Detection was performed using LumiBest ECL Luminescent Liquid (Share-Bio, Shanghai, China).

### Measurement of intracellular Ca^2+^ levels

A549 cells were seeded in 12-well plates, pre-treated with various reagents, and subsequently infected with NDV. A working solution of Fluo-4 AM (5 μM) was prepared using blank DMEM with 0.04% Pluronic® F-127 (AAT Bioquest, Pleasanton, USA). Then, 1 mM probenecid (AAT Bioquest, Pleasanton, USA) was added to the dye working solution to minimize dye leakage. After 1 h of incubation in the dark, the cells were washed three times with PBS then either directly observed using fluorescence microscopy at a wavelength of 488/525 nm (Olympus, Tokyo, Japan) or subjected to FCM to measure the fluorescence intensity.

### Virus adsorption and internalization assay

Cells were pre-treated at 4°C for 1 h and subsequently infected with NDV at a MOI of 50 for 1 h at 4°C. Following infection, the cells were washed three times with cold PBS to eliminate any unbound viruses. Total RNA was isolated using TRIzol reagent and reverse transcribed into complementary DNA. NDV NP mRNA levels were quantified by RT-qPCR. To visualize NDV attachment to the host plasma membrane, virions were fluorescently labeled with the lipophilic dye DiD. NDV stock (50 MOI) was prepared in serum-free medium and mixed 1:1,000 with 10 µM DiD (final concentration). Gently invert the tube to mix, then incubate at room temperature in the dark for 30 min to allow DiD to integrate into the viral envelope.The suspension was passed through a 0.45 µm filter to remove dye aggregates. Unincorporated DiD was eliminated by three consecutive washes in 50-kDa Amicon Ultra centrifugal filters (4°C, 3,000 × g, 10 min each), resuspending the retained virus to the original volume with serum-free DMEM after each wash. DiD-labeled NDV was added to chilled A549 cells and incubated at 4°C for 1 h to permit surface adsorption while blocking endocytosis. After three gentle washes with ice-cold PBS to remove unbound virions, the cells were fixed with 4 % paraformaldehyde for 10 min at room temperature and rinsed thoroughly with PBS. Without permeabilization, the intact monolayers were immediately imaged by confocal laser-scanning microscopy. DiD fluorescence was used to quantify virion density on the plasma membrane.

Following virus adsorption, the virus was aspirated and replaced with pre-warmed medium. Cells were then incubated at 37°C with 5% CO₂ for 1 h to allow virus internalization. Residual surface-bound virions were stripped by a 30-s gentle rinse with ice-cold acidic PBS (pH 3.0), followed by three washes with standard PBS. Intracellular viral genomes were quantified by RT-qPCR to assess internalization efficiency between groups.Alternatively, virions were labeled with DiD and subjected to the same protocol. Following internalization, cells were fixed directly with 4 % paraformaldehyde for 10 min and imaged by confocal microscopy to visualize DiD fluorescence.

### Blocking assay

Before infection, NDV virions were pre-incubated with escalating concentrations of Annexin V or anti-PS antibody for 2 h at room temperature to block surface-exposed PS. Cells were then infected, and viral replication was evaluated at 12 hpi by RT-qPCR and Western blot.

### Statistical analysis

Statistical analysis was performed using GraphPad Prism. All bar graph data are presented as the mean ± standard error of the mean (SEM) from a minimum of three independent replicates. Statistical significance is denoted by * for *p* < 0.05 and ** for *p* < 0.01. Data were subjected to Student’s t-test for pairwise comparisons and analysis of variance (ANOVA) for multiple group comparisons.

## Result

### Plasma-membrane PS is critical for ndv budding from the host cell

**Figure 1.**
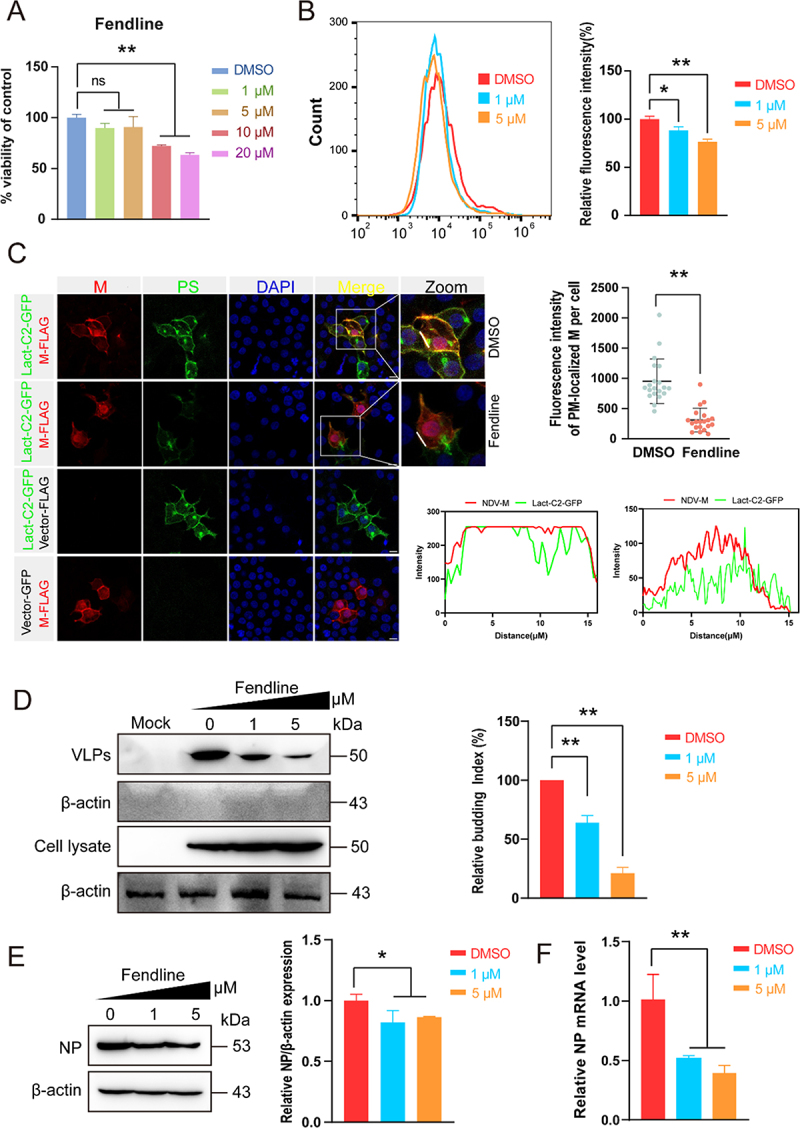
Plasma-membrane PS is critical for NDV budding from the host cell (A) A549 cells seeded in 96-well plates were treated with the indicated concentrations of Fendiline for 24 h, and viability was measured with the CCK-8 assay (*n* = 3 wells per group). (B) After 48 h of Fendiline treatment, cells were permeabilized, labelled with Annexin V – fluorochrome and total ps was quantified by flow cytometry. (C) HEK-293T cells were co-transfected with plasmids encoding M and the PS probe Lact-C2-GFP. At 24 h post-transfection (hpt) Fendiline was added; colocalization of M (red) and PS (green) was assessed by confocal microscopy. Scale bar, 10 µm. (D) HEK-293T cells transfected with M alone were treated with DMSO or Fendiline (1 or 5 µM) for 48 h. VLPs and cell lysates were harvested and analysed by Western blot. The budding index (bottom) was calculated as M in VLPs/(M in VLPs + M in lysate) normalized to DMSO. (E and F) A549 cells were pre-treated with Fendiline or vehicle, infected with NDV (MOI = 0.1) and analysed 24 hpi by Western blot (E) and RT-qPCR for NP mRNA(F). Data are presented as mean ± sem of three independent experiments. Statistical significance was determined by one-way anova test; *p* < 0.05, *p* < 0.01 vs. DMSO control group.

The matrix (M) protein of NDV orchestrates viral assembly and budding. Ectopic expression of M alone is sufficient to trigger the formation and release of virus-like particles (VLPs), indicating that M autonomously engages the plasma membrane and drives membrane curvature and scission [28]. Furthermore, the M protein surface is positively charged, which may enable it to interact with negatively charged PS present in the cell membrane. Fendline is a small-molecule drug that effectively reduces PS levels in the plasma membrane [29]. Here, we utilized Fendline to deplete PS levels in cells. First, we evaluated Fendiline cytotoxicity in HEK-293T cells. After 48 h of exposure, cell viability remained ≥90 % at concentrations up to 5 µM (Figure 1(A)); this dose was therefore established as the nontoxic upper limit. Next, HEK-293T cells were treated with vehicle (DMSO) or Fendiline (1 or 5 µM) for 48 h. Flow-cytometric quantification of Annexin V binding revealed a concentration-dependent reduction in total PS levels: 11.68% at 1 µM (*p* < 0.05) and 23.32% at 5 µM (*p* < 0.01) relative to DMSO controls (Figure 1(B)). Confocal imaging of permeabilized A549 cells confirmed these findings, showing a marked decrease in membrane-associated PS fluorescence after Fendiline treatment (Figure S1(A)).

To visualize the spatial relationship between PS and the NDV matrix (M) protein, we generated the PS biosensor Lact-C2-GFP. HEK-293T cells were co-transfected with plasmids encoding M and Lact-C2-GFP, treated ± Fendiline for 24 h, and analyzed by confocal microscopy. Confocal imaging revealed extensive colocalization of PS and the M protein at the plasma membrane in control cells, whereas Fendiline treatment markedly reduced their spatial overlap. Quantitative analysis confirmed a significant decrease in membrane-associated M protein upon Fendiline treatment (Figure 1(C)). In DMSO-treated cells, M protein was predominantly localized to the nucleus and the plasma membrane, whereas Fendiline elicited a pronounced cytoplasmic redistribution of M, indicating that PS depletion impairs M anchoring to the plasma membrane. To determine the functional consequence of PS depletion on virion release, we performed a VLP budding assay. Cells expressing M alone were exposed to Fendiline (1 or 5 µM) for 48 h. Western blot analysis of concentrated supernatants revealed a progressive reduction in VLP-associated M: relative budding efficiency decreased to 60% at 1 µM and 25% at 5 µM compared with DMSO controls (*p* < 0.01; Figure 1(D)). To determine whether Fendiline suppresses NDV replication and dissemination, we quantified NP expression by RT-qPCR and Western blot. Fendiline conferred a dose-dependent inhibition: relative to vehicle-treated controls, NP mRNA and protein levels were significantly reduced at all tested concentrations (*p* < 0.01) (Figure 1(E,F)). We next assessed whether Fendiline impairs NDV adsorption, internalization, or early replication. No significant differences were detected between Fendiline-treated and vehicle-treated cells at any step examined (Figure S1(B-F)). Moreover, PS was detected in the Concentrated VLPs (Figure S1(G)).

### PS is a key phospholipid in ndv infection

**Figure 2.**
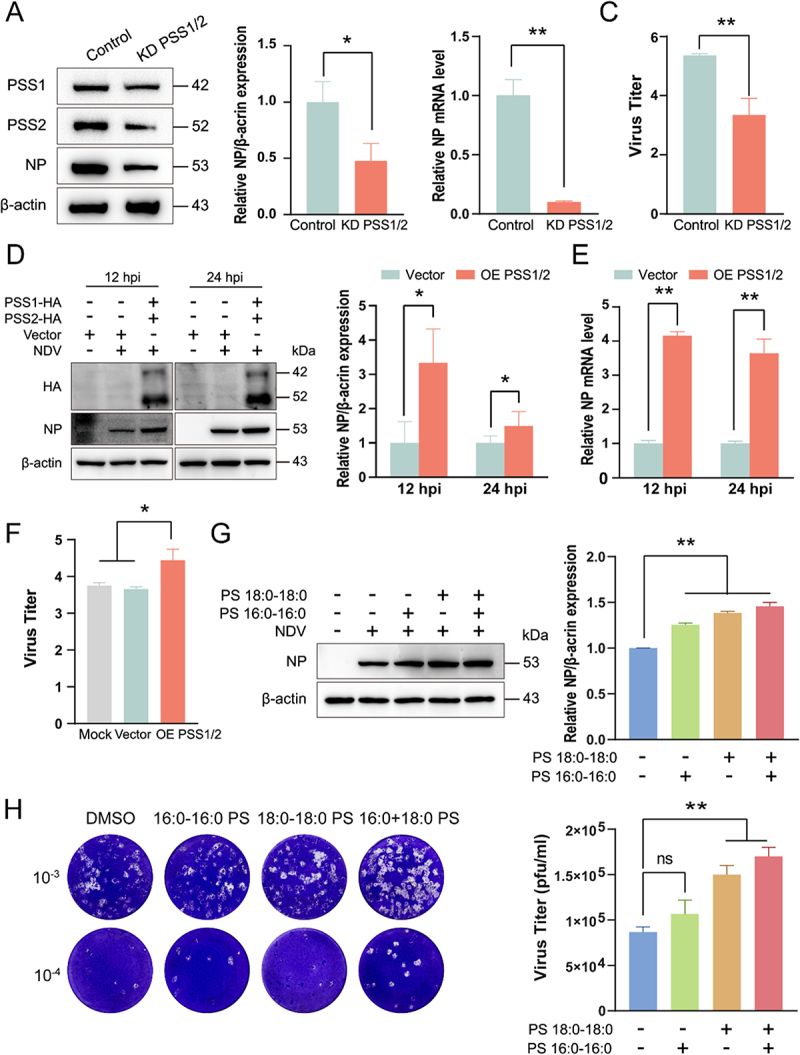
PS is a key phospholipid in NDV infection. (A–C) A549 cells transfected with CRISPR/Cas9 knockout plasmids targeting PSS1 or PSS2. were infected with NDV (MOI = 0.1). NP mRNA and protein were measured at 12 hpi by RT-qPCR (A) and Western blot (B); viral titres were determined by TCID₅₀ (C). (D–F) Over-expression of PSS1/2 was achieved by plasmid transfection (24 h) before infection (MOI = 0.1); NP mRNA (D), protein (E) and titres (F) were analysed as above. (G and H) Cells were pre-incubated with the indicated concentrations of exogenous PS (20 μM) for 2 h, infected with NDV (MOI = 0.1) and maintained in PS-containing medium. NP protein and infectious titer were assessed at 12 hpi by western blot (G) and plaque assay (H).

To assess the contribution of membrane PS to NDV replication, we genetically modulated the expression of PSS1 and PSS2—the rate-limiting enzymes of PS biosynthesis. Silencing PSS1/2 markedly attenuated viral propagation: at 12 hpi, NP protein was reduced by 57 % (Figure 2(A)), and both NP mRNA levels and infectious titers dropped significantly (*p* < 0.01) (Figure 2(B,C)). Conversely, overexpression of PSS1/2 potentiated replication, increasing NP protein by 281 % at 12 hpi and 50 % at 24 hpi (Figure 2(D)), accompanied by elevated NP mRNA and viral yields (*p* < 0.05) (Figure 2(E,F)). Although metabolic enzymes typically do not directly regulate viral replication, they can indirectly influence viral replication through their metabolites. To directly test the impact of PS on NDV replication, we exogenously supplemented cultures with defined PS species. Addition of 16:0–16:0 PS or 18:0–18:0 PS individually enhanced viral propagation, whereas their combined supplementation produced a synergistic increase in replication (Figure 2(G,H)). To assess specificity, we supplemented cultures with phosphatidylcholine (PC) or phosphatidylethanolamine (PE); neither lipid altered NDV replication (Figure S2(A-D)). These results establish cellular PS abundance as a critical determinant of NDV replication efficiency.

### Surface-exposed PS on NDV mimics an “eat-me” signal

**Figure 3.**
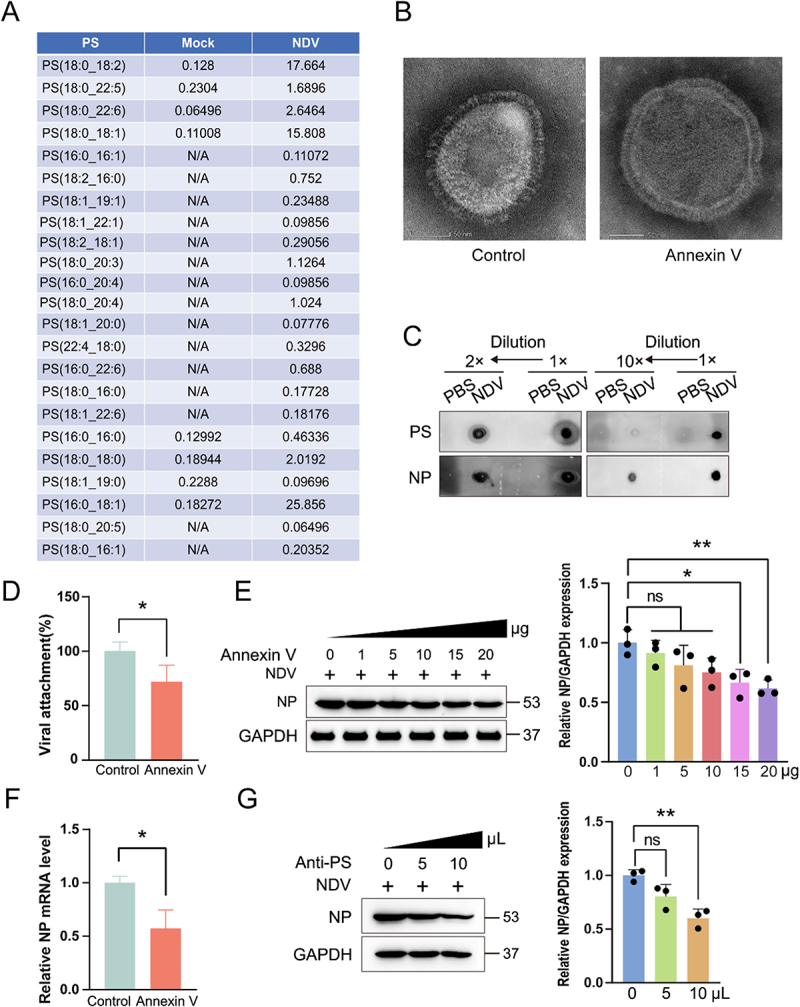
Surface-exposed PS on NDV mimics an “eat-me” signal. (A) Virions purified from 24 h culture supernatants by sucrose-gradient centrifugation were subjected to LC-MS/MS for PS detection. (B) Negative-stain TEM shows intact NDV (left) and virions pre-blocked with Annexin V (right). (C) dot-blot of serially diluted purified virions probed with anti-PS or anti-NP antibody. (D) A549 cells were incubated with NDV (50 MOI) pre-treated with Annexin V (20 µg); adsorbed genomes were quantified by RT-qPCR. (E) Dose-dependent inhibition of infection (0.1 MOI) by Annexin V (20 µg) was assessed by Western blot for NP (12 hpi). (F) Virions were pre-incubated with Annexin V (20 µg) before infection (0.1 MOI); NP mRNA was measured at 12 hpi. (G) Virions were pre-incubated with increasing concentrations of anti-PS antibody for 2 h before infection (0.1 MOI); NP protein was analysed by Western blot.

PS functions as a universal “eat-me” signal that enables enveloped viruses to masquerade as apoptotic bodies and engage PS receptors on host cells. To investigate the presence of PS in NDV virions and its potential role in viral infection, we isolated purified NDV virions by sucrose gradient centrifugation. Subsequently, we detected PS in the NDV virions using LC-MS/MS. Figure 3(A) illustrates the abundance of PS in the NDV virions. Negative-stain transmission electron microscopy images revealed the intact structure and morphology of the NDV particles, with a distinctly visible envelope layer (Figure 3(B)). Dot-blot analysis further demonstrated robust PS exposure on the virion surface (Figure 3(C)). To assess the relevance of virion-associated PS, purified NDV was pre-incubated with Annexin V – a high-affinity PS-binding protein – prior to infection. Annexin V neutralization resulted in a significant reduction in viral adsorption (*p* < 0.05) (Figure 3(D) and S3(A)) and conferred a dose-dependent suppression of NDV replication (Figure 3(E,F)). Parallel experiments using anti-PS antibodies recapitulated this inhibitory effect in a concentration-dependent manner (Figure 3(G)). Collectively, these data establish that NDV displays PS on its envelope as an apoptotic mimic, a critical determinant for efficient cell entry and subsequent replication.

### NDV infection drives TMEM16F-dependent PS externalization

**Figure 4.**
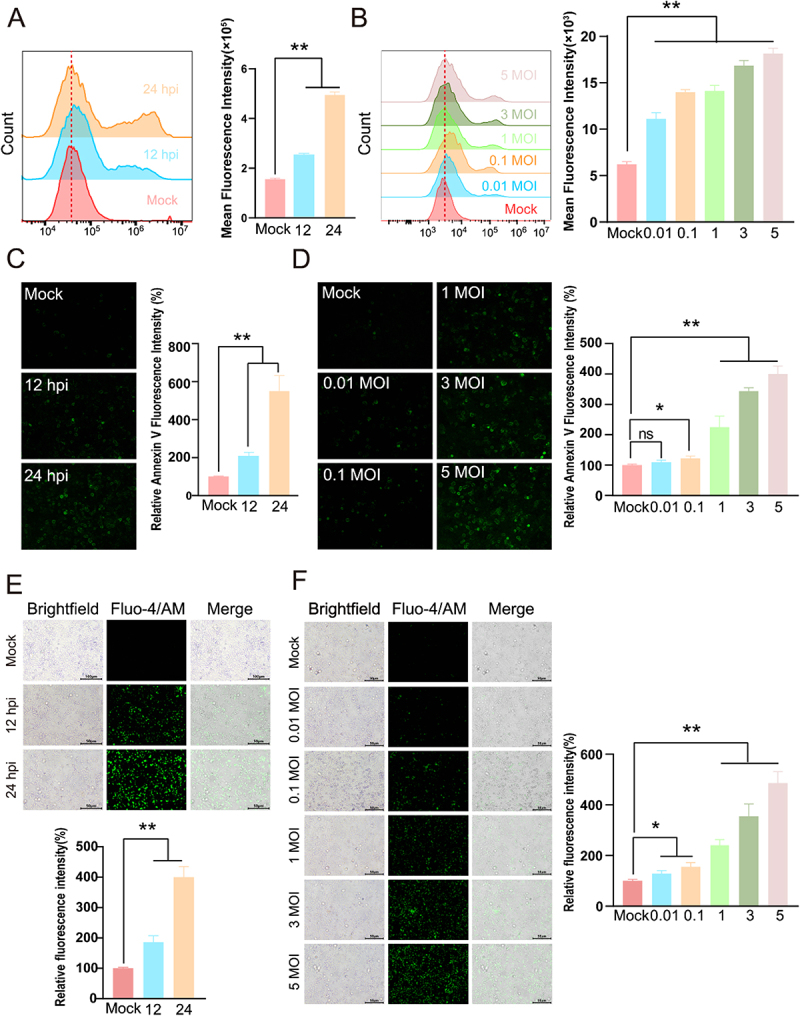
NDV infection drives TMEM16F-dependent PS externalization. (A–D) A549 cells were infected with NDV or left uninfected. Surface PS was quantified at the indicated time points by flow cytometry (A, B) and fluorescence microscopy (C, D). In A and C, infections were performed at an MOI of 1 and sampled at 12 and 24 hpi; in C and D, infections were performed at an MOI of 0.01, 0.1, 1, 3, and 5 and sampled at 12 hpi. (E and F) Cytosolic Ca^2 +^ was monitored with Fluo-4 AM under identical conditions.

Engagement of enveloped viruses with host cell receptors has been shown to trigger PS externalization at the plasma membrane. During assembly and budding, these viruses incorporate PS into their envelopes, thereby acquiring an apoptotic-mimicry signature that facilitates entry. Consistent with this paradigm, NDV was expected to bud as PS-positive virions. Using flow cytometry and quantitative fluorescence imaging, we monitored PS exposure on infected cells over time and across a range of multiplicities of infection. NDV infection elicited a robust, time- and dose-dependent increase in cell-surface PS externalization (*p* < 0.01) (Figure 4(A–D)). The precise molecular mechanism by which NDV induces PS exposure, however, remains to be elucidated. To determine whether PS externalization is confined to infected cells, we infected A549 cells with NDV-mCherry and quantified surface PS by flow cytometry. PS exposure was exclusively detected in mCherry-positive virus-infected cells, confirming that PS externalization is a selective consequence of NDV infection ((Figure S4(A)).

Scramblases, specifically TMEM16F, play a significant role in PS externalization. To determine whether NDV engages this pathway, we monitored intracellular Ca^2 +^ dynamics in A549 cells following infection. Fluo-4 AM imaging revealed a robust, time- and dose-dependent elevation in cytosolic Ca^2 +^, reflected by both increased fluorescence intensity and a higher proportion of Ca^2 +^ -positive cells (Figure 4(E,F)). These data indicate that NDV infection creates the Ca^2 +^ milieu required for TMEM16F activation and subsequent PS exposure.

### Ca^2+^ -activated TMEM16F is implicated in the NDV-induced externalization of ps

**Figure 5.**
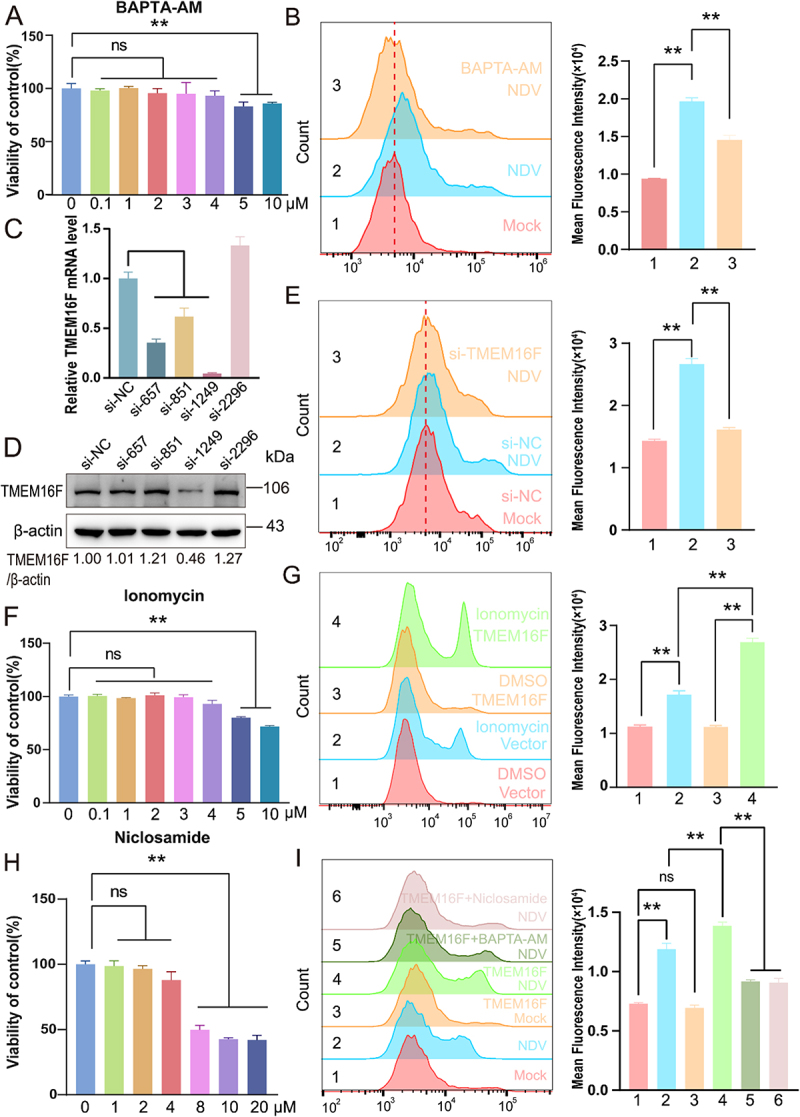
Ca^2+^-activated TMEM16F is implicated in the NDV-induced externalization of PS. (A) CCK-8 assays on A549 cells were performed to evaluate the BAPTA-AM toxicity. (B) Cells were either pre-treated with BAPTA-AM (4 μM) or mock-treated, following infection of NDV (1 MOI). Extracellular PS levels were analyzed by FCM (12 hpi). (C and D) Efficiency of siRNA interference with TMEM16F expression was evaluated by RT-qPCR and western blot. (E) Extracellular PS levels after interference with TMEM16F expression levels. Data are presented as mean ± sem (*n* = 3). Significance was analyzed by unpaired Student’s t-test (*p* < 0.01). (F) CCK-8 assays on A549 cells were performed to evaluate Ionomycin toxicity. (G) A549 cells were transfected with a plasmid encoding TMEM16F for 24 h, then treated with Ionomycin (4 μM) or left untreated. Extracellular PS levels were then measured using FCM. (H) CCK-8 assays on A549 cells were performed to evaluate Niclosamide toxicity. (I) A549 cells were transfected with a plasmid encoding TMEM16F for 24 h, followed by infection with NDV (1 MOI). Subsequently, the cells were treated with either BAPTA-AM or Niclosamide(4 μM) or left untreated. Extracellular PS levels were then measured using FCM. Data are presented as mean ± SEM (*n* = 3). Significance was analyzed by unpaired Student’s t-test (*p* < 0.01).

To delineate the molecular basis of NDV-triggered PS externalization, we focused on the Ca^2 +^ -activated scramblase TMEM16F.Specifically, we explored the effect of Ca^2+^ on PS externalization in NDV-infected A549 cells. Treatment with the intracellular Ca^2+^ chelator BAPTA-AM significantly decreased NDV-induced PS externalization (*p* < 0.01) (Figure 5(A,B)). Consistent with this observation, siRNA-mediated silencing of TMEM16F markedly diminished PS externalization in NDV-infected cells (*p* < 0.01) (Figure 5(C–E)). Conversely, ectopic expression of TMEM16F alone did not alter basal PS levels, yet substantially potentiated ionomycin-induced PS externalization (Figure 5(F,G)). Importantly, TMEM16F overexpression amplified NDV-triggered PS externalization (*p* < 0.01), and this augmentation was reversed by BAPTA-AM (*p* < 0.01) and by the TMEM16F inhibitor Niclosamide (*p* < 0.01) (Figure 5(H,I)).Collectively, these data establish that TMEM16F functions as the principal Ca^2 +^ -dependent scramblase responsible for PS externalization in NDV-infected cells, and that NDV hijacks host Ca^2 +^ signaling to activate TMEM16F and thereby promote PS exposure.

### The HN protein is essential for NDV-triggered ps externalization

**Figure 6.**
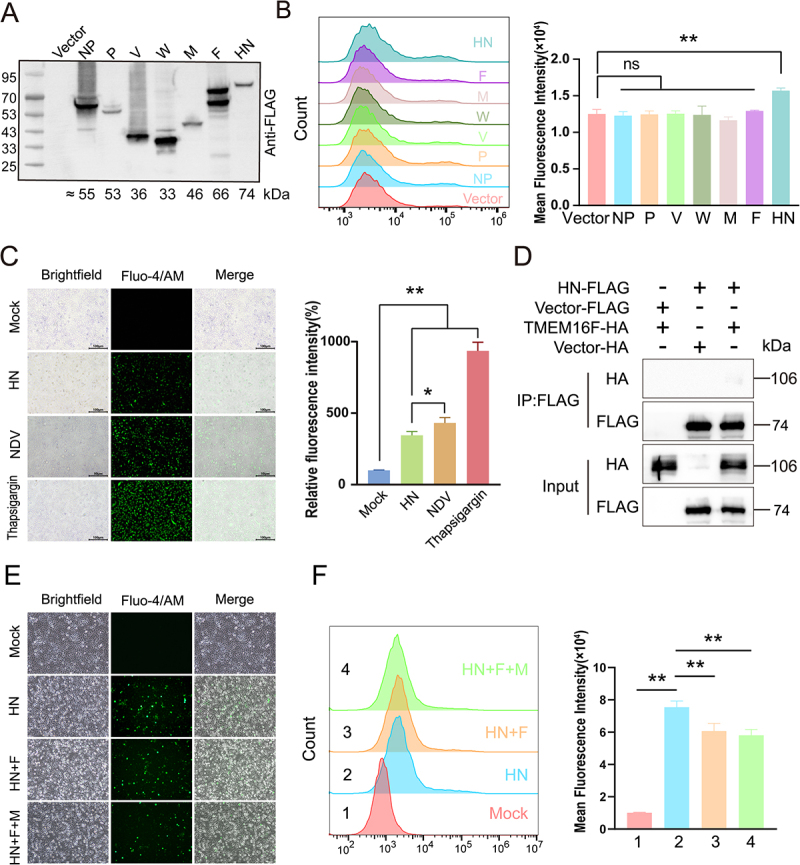
The HN protein is essential for NDV-triggered PS externalization. (A) Expression of NDV structural and non-structural proteins were analysed by Western blot. (B) A549 cells transfected with plasmids encoding individual NDV proteins for 36 h were analysed for surface PS by flow cytometry. (C) Cells transfected with HN, infected with NDV, or treated with endoplasmic reticulum stress inducer thapsigargin (4 µM) were loaded with Fluo-4 am and imaged. (D) Co-immunoprecipitation of HA-TMEM16F and Flag-HN after co-transfection in HEK-293T cells. (E and F) Cells expressing HN, F or M were loaded with Fluo-4 AM and analyzed by fluorescence microscopy (E) and flow cytometry (F).

NDV infection triggers robust PS externalization in A549 cells. To pinpoint the viral determinant responsible for this event, we systematically expressed each structural and non-structural NDV protein and assessed their individual capacities to drive PS exposure. Among the viral proteins tested, only HN significantly induced PS externalization (Figure 6(A,B)). Real-time Ca^2 +^ imaging revealed that ectopic expression of HN elevated cytosolic Ca^2 +^ levels, albeit to a lesser extent than whole-virus infection (Figure 6(C)). Thapsigargin, used as a positive control for ER Ca^2 +^ release, produced the highest Ca^2 +^ signal, confirming assay sensitivity. These data indicate that HN alone is sufficient to trigger Ca^2 +^ accumulation and, consequently, TMEM16F activation. To test whether HN interacts directly with TMEM16F, we performed co-immunoprecipitation assays. Despite robust expression of both proteins, no association between HN and TMEM16F was detected (Figure 6D). Collectively, our findings support a model in which NDV HN indirectly activates TMEM16F by elevating intracellular Ca^2 +^, thereby promoting PS externalization without requiring a direct protein – protein interaction.

Although ectopic expression of NDV HN alone elevated cytosolic Ca^2 +^, the amplitude remained lower than that observed during whole-virus infection. We therefore asked whether co-expression of additional viral proteins – specifically F, or the combination M+F – would synergize with HN to fully activate TMEM16F and maximize PS externalization. Surprisingly, neither the HN+F nor the HN+F+M combinations increased Ca^2 +^ influx beyond the level achieved by HN alone; in fact, signal intensity declined slightly (Figure 6(E,F)). The reason for the slightly reduced signal in the co-expression groups remains unclear but may involve complex interactions between viral proteins or with host factors that modulate Ca^2 +^ signaling, a fascinating aspect that warrants further investigation.

### PS externalization enhances NDV replication

**Figure 7.**
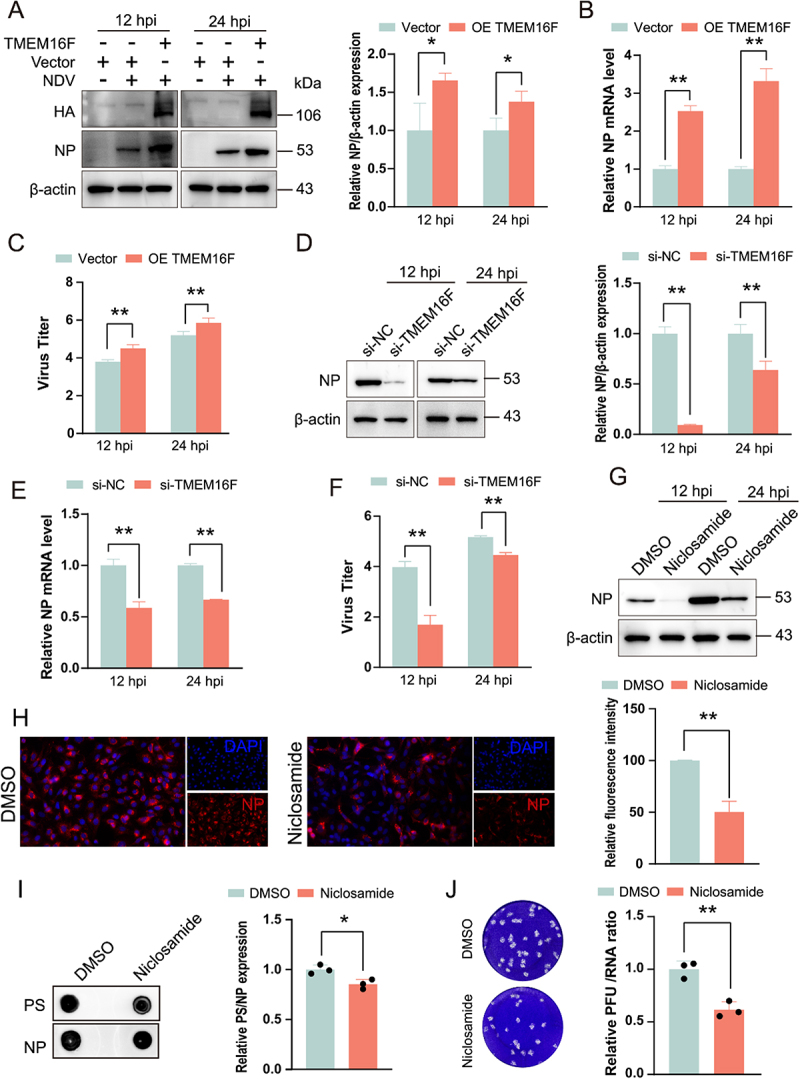
PS externalization enhances NDV replication. (A-C) A549 cells over-expressing TMEM16F were infected with NDV (MOI = 0.1); NP mRNA, protein and viral titres were determined at 12 and 24 hpi. (D–F). TMEM16F was silenced with siRNA prior to infection and analysed as above. (G and H). Cells were pre-treated with Niclosamide (4 µM) or vehicle before infection; replication was monitored by immunofluorescence (G) and western blot (H). (I) Virions released from Niclosamide- or mock-treated cells (24 hpi) were concentrated, NP expression levels equalised, and PS content determined by dot-blot with anti-PS antibodies. (J) Virions released from Niclosamide- or mock-treated cells (24 hpi) were collected and viral genomes were quantified by qRT-PCR. After normalizing to equal genome copies, specific infectivity (PFU/genome copy) was determined by plaque assay.

NDV-induced PS externalization is executed by the host Ca^2 +^ -activated scramblase TMEM16F. To determine whether this lipid remodeling supports viral propagation, we genetically and pharmacologically manipulated TMEM16F activity in A549 cells. Our findings indicate that the overexpression of TMEM16F significantly enhances NDV replication. Specifically, the levels of NP increased by 44% at 12 hpi and by 77% at 24 hpi (Figure 7(A)). Additionally, mRNA expression levels of NP and the viral titer were significantly elevated (*p* < 0.01) (Figure 7(B,C)). Conversely, the downregulation of TMEM16F resulted in a marked inhibition of NDV replication, with NP protein levels decreasing by 85% at 12 hpi and by 34% at 24 hpi (Figure 7(D)). Furthermore, both mRNA levels of NP and the viral titer were significantly reduced (*p* < 0.01) (Figure 7(E,F)). Notably, treatment with Niclosamide significantly suppressed NDV replication (Figure 7(G,H)), with the protein level and fluorescence intensity of NP in the Niclosamide-treated group decreasing by an average of 52% compared to the DMSO group (*p* < 0.01). Subsequent experiments utilizing NDV-GFP under consistent conditions confirmed that Niclosamide significantly inhibited NDV replication (Figure S5(A)). We next assessed whether Niclosamide impairs NDV adsorption, internalization, or early replication. Niclosamide does not interfere with NDV attachment or the early phase of replication; instead, it impedes viral internalization (Figure S5(B-D)). Treatment with the Ca^2 +^ inducer Ionomycin failed to further stimulate NDV replication, suggesting that the Ca^2 +^ mobilization elicited by NDV itself is already sufficient to fully activate TMEM16F-mediated PS externalization (Figure S5(E)). To demonstrate that TMEM16F knockdown or inhibition attenuates NDV replication specifically via reduced PS content in the viral envelope, we first quantified PS levels in virions by dot-blot. Particles harvested from Niclosamide-treated cells exhibited markedly lower PS abundance than those from DMSO group (Figure S7(I)). Next, viral genome copies were determined by RT-qPCR, and virions were normalized to identical genome numbers prior to plaque assay. The ratio of infectious PFU to genome copies – an indicator of specific infectivity – was significantly diminished in the Niclosamide group relative to the mock-treated group(Figure S7(J)). Collectively, these data substantiate our hypothesis that TMEM16F depletion or inhibition compromises NDV infectivity through altered PS incorporation into the viral envelope.

### TYRO3 and TIM-4 function as ps receptors to mediate NDV entry into A549 cells

**Figure 8.**
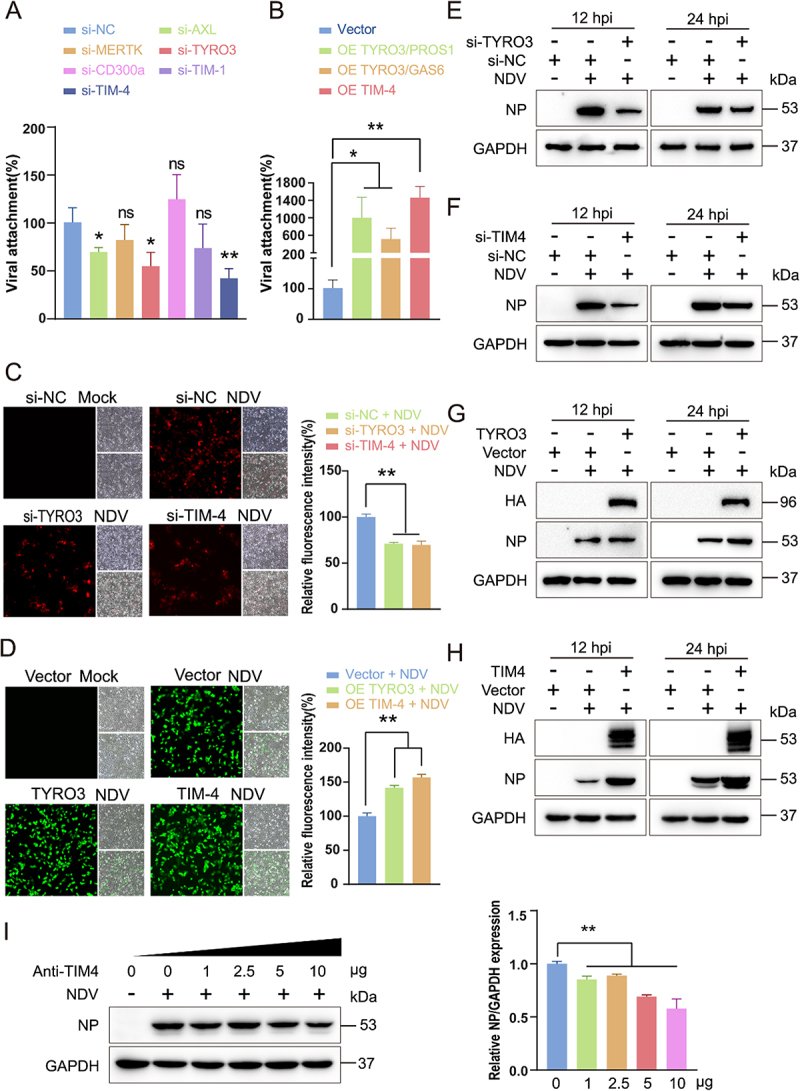
TYRO3 and TIM-4 function as PS receptors to mediate NDV entry into A549 cells (A) A549 cells transfected with the indicated siRnas were infected with NDV (MOI = 50). NP mRNA was quantified at 1 hpi to assess entry. (B) Over-expression of TYRO3/GAS6, TYRO3/PROS1 or TIM-4 were infected with NDV (MOI = 50). Data are presented as mean ± SEM of three independent experiments (*n* = 3). Statistical significance was determined by one-way anova test; *p* < 0.05, *p* < 0.01 vs. vector control group. NP mRNA was quantified at 1 hpi to assess entry. (C and D) fluorescence intensity of RFP-NDV (C) or GFP-NDV (D) was quantified after silencing or over-expressing TYRO3/TIM-4. (E–H) NP protein levels were measured by western blot under identical conditions. (I) A549 cells were pre-incubated with increasing concentrations of anti-TIM-4 antibody for 1 h before infection; NP protein was analyzed at 12 hpi.

PS receptors – including TIM-1, TIM-3, TIM-4, CD300A, MFG-E8, AXL, TYRO3, and MERTK – serve as critical entry cofactors for a broad range of enveloped viruses. To determine which of these receptors recognize NDV virions as apoptotic mimics, we first profiled their basal expression in A549 cells. With the exception of TIM-3, all PS receptors were robustly transcribed (Figure S6(A)). Kinetic analysis across the course of infection revealed a significant late-phase induction of TIM-1, TIM-4 and CD300a transcripts (*p* < 0.05), whereas AXL, MERTK and TYRO3 were modestly up-regulated and MFG-E8 levels remained unchanged (Figure S6(B–G)). To pinpoint the receptors that directly engage PS on the NDV envelope, we selectively modulated their expression and quantified viral adsorption. siRNA-mediated knockdown of either TYRO3 or TIM-4 markedly impaired NDV attachment (*p* < 0.05) (Figure 8(A), S6H and S6J), while ectopic overexpression of the same receptors significantly enhanced viral binding (*p* < 0.05; Figure 8(B0, S6I and 6K). Collectively, these data identify TYRO3 and TIM-4 as the principal PS receptors mediating NDV adsorption, warranting further mechanistic investigation. Leveraging the demonstrated roles of TYRO3 and TIM-4 in NDV attachment, we quantified reporter fluorescence to interrogate their impact on the complete replication cycle. ImageJ-based analysis of fluorescent recombinant NDV-infected cells revealed that CRISPR-mediated silencing of either receptor markedly attenuated fluorescence output (*p* < 0.01), whereas overexpression significantly promoted it (*p* < 0.01) (Figure 8(C,D)). We next dissected the functional roles of TYRO3 and TIM-4 in NDV replication by western blotting and RT-qPCR. Genetic silencing of either receptor markedly suppressed infection: NP protein abundance was significantly reduced at 12 and 24 hpi (Figure 8(D,F), S7(A,B)), and NP mRNA levels declined by 12 hpi (P<0.01) (Figure S7(C) and Figure S7(D)). Conversely, overexpression of TYRO3 or TIM-4 potentiated NDV replication (Figure 8(E)), evidenced by elevated NP protein at 12 and 24 hpi (Figure 8(G,H), S7(E,F)) and increased NP gene transcription at 12 hpi (Figure S7(G,H)). To confirm that ectopic TYRO3 and TIM-4 exceed endogenous levels and are properly delivered to the plasma membrane, we performed confocal microscopy. Because no TYRO3 antibody suitable for immunofluorescence is currently available, we appended an HA tag to the exogenous construct and co-stained the plasma membrane with ATP1A1. The resulting images revealed the plasma-membrane localization of ectopically expressed TYRO3 (Figure S7(I)). TIM4 antibody demonstrated markedly increased surface expression and membrane localization upon overexpression (Figure S7(J)).

TIM-4 displayed the strongest pro-viral phenotype among all PS receptors examined. Neutralizing TIM-4 with a monoclonal antibody produced a dose-dependent suppression of NDV replication (Figure 8(I)). To determine whether TIM-4 functions independently of canonical sialic-acid engagement, we enzymatically removed cell-surface sialic acids with neuraminidase. Neuraminidase treatment attenuated NDV infection in a concentration-dependent manner; nevertheless, TIM-4 overexpression partially restored viral yields (Supplementary Figure S7(K)), confirming that TIM-4 can compensate for reduced sialic-acid binding. Importantly, neither TYRO3 nor TIM-4 overexpression enhanced avian influenza virus replication; instead, both receptors modestly suppressed it (Supplementary Figure S7L), indicating that AIV does not exploit TYRO3/TIM-4-mediated PS recognition. Collectively, these data establish TYRO3 and TIM-4 as selective PS receptors that enable A549 cells to recognize and internalize NDV virions through apoptotic-mimicry-dependent mechanisms.

## Discussion

Viruses have evolved intricate strategies to co-opt host cellular machinery, and the manipulation of host lipid metabolism is emerging as a central theme in virus-host interactions [30]. Viral infections profoundly alter cellular lipid distribution. During entry, viruses commandeer existing lipids and signaling pathways for transport. Subsequent viral gene expression triggers intensive reprogramming of lipid synthesis and reallocation to fuel replication, assembly, and release [31]. In this study, we unveil a sophisticated, two-pronged mechanism by which NDV exploits host PS to enhance its infectivity, impacting both the late stages of viral budding and the early stages of entry into new cells. Our results first establish a critical role for PS in the assembly and budding of NDV. The viral M protein, a key orchestrator of virion formation, was found to colocalize with PS at the plasma membrane. Depletion of cellular PS with Fendiline or genetic knockout of PS synthases (PSS1/2) disrupted the membrane localization of the M protein and significantly impaired the release of virus-like particles (VLPs) and infectious virions (Figure 1, 2). This is consistent with the known polybasic nature of the M protein surface, which likely facilitates electrostatic interactions with the negatively charged headgroup of PS, thereby anchoring the assembly process to specific membrane domain [32]. Indeed, we confirmed that M-driven VLPs are themselves enriched in PS (Figure S1(G)). These findings align with recent studies on other paramyxoviruses, such as measles and Nipah viruses, where M protein association with PS is crucial for triggering membrane deformation and virion assembly [33]. Our data extend this concept to NDV, highlighting that the availability of PS in the host membrane is a key determinant of efficient viral production, however, the exact biophysical mechanisms governing this interaction remain to be determined in future studies.

Beyond its role in budding, our study provides the first direct evidence that NDV employs “apoptotic mimicry” as a parallel pathway to infect host cells. This mechanism is initiated by the virus itself. We demonstrated that NDV infection, specifically through the expression of its HN glycoprotein, triggers a significant influx of intracellular Ca^2+^ (Figure 6). This elevated cytosolic Ca^2+^ directly activates the host phospholipid scramblase TMEM16F, resulting in the rapid externalization of PS to the surface of infected cells (Figure 4, 5). Consequently, progeny virions that bud from these PS-rich membrane regions acquire a PS-laden envelope, effectively masquerading as apoptotic bodies [31]. Viruses employ distinct strategies to acquire PS. For instance, EBOV buds PS-enriched envelopes from plasma membrane lipid rafts [34,35], while vaccinia virus incorporates PS from endoplasmic reticulum (ER) fragments [36], and dengue virus buds from the ER itself [37]. Notably, while our data demonstrate that NDV HN protein expression elevates intracellular Ca^2 +^ (activating TMEM16F), the precise molecular mechanism triggering this Ca^2 +^ influx remains unresolved and requires future resolution. Critically, this pathway enables TMEM16F-mediated PS exposure on infected cells, ultimately facilitating NDV budding and acquisition of a PS-rich envelope. Although the exact mechanisms of viral PS acquisition vary, this Ca^2 +^ elevation strategy is conserved across diverse viruses [38–42], indicating our findings illuminate a broadly exploitable host pathway. Furthermore, scramblases such as XKR8 are known mediators of PS exposure during physiological processes (platelet activation, apoptosis), our findings do not exclude its potential involvement in NDV-mediated PS externalization, warranting future study.

Multiple lines of evidence establish apoptotic mimicry as a viable viral propagation strategy. Blocking NDV surface PS with Annexin V or anti-PS antibodies significantly reduced viral adsorption and replication (dose-dependent), confirming PS’s essential role. TYRO3 and TIM-4 were identified as NDV-recognizing PS receptors: TYRO3 interference reduced NDV adsorption by 55%, while TIM-4 interference reduced adsorption by 42%. Conversely, TYRO3 overexpression increased NDV NP levels to 133%, whereas TIM-4 overexpression increased NP levels to 395%. Anti-TIM4 antibody treatment also dose-dependently inhibited replication. Neuraminidase-mediated sialic acid removal inhibited NDV infection, but TIM-4 overexpression partially rescued replication, confirming PS mimicry as key for NDV cell-to-cell spread. Notably, this contrasts with avian influenza virus, where TYRO3/TIM-4 overexpression inhibits replication. Furthermore, PRRSV is recognized by TIM-1 and TIM-4 [43], ASFV by MFG-E8, TIM-4, and AXL [22,23], SARS-CoV-2 by TIM-1, TIM-4, and AXL [44], and EBOV by TIM-1, AXL, and TYRO3 [24–26]. This raises a critical question: Why do different PS receptors exhibit variable abilities to enhance viral entry? Likely, heterogeneity in viral envelope PS distribution/concentration influences receptor binding specificity and cellular tropism. Comprehensive studies are needed to resolve these mechanisms.

In conclusion, this study reveals how NDV employs apoptotic mimicry by hijacking the HN-Ca^2 +^ -TMEM16F pathway to remodel the host plasma membrane, yielding PS-enriched progeny virions. These exploit TYRO3 and TIM-4 receptors to enhance cellular entry, operating parallel to the traditional sialic acid pathway. This PS-dependent mechanism represents a novel, promising target for host-directed antiviral strategies against Newcastle disease.

## Supplementary Material

Figure S2.tif

Figure S6.tif

Figure S1.tif

Figure S5.tif

Figure S3.tif

Figure S7.tif

Figure S4.tif

Supplementary Material 0902 final.docx

## Data Availability

All original data are stored in the Science Data Bank repository (https://doi.org/10.57760/sciencedb.20177) [45] under a CC-BY-4.0 license.
