# Supplementary figures and images for: Newcastle disease virus acquires phosphatidylserine through the budding process to enhance infectivity
Source: Virulence. 2025 Oct 28;16(1):2580150. doi: 10.1080/21505594.2025.2580150 (PMC12604639; doi:10.1080/21505594.2025.2580150)

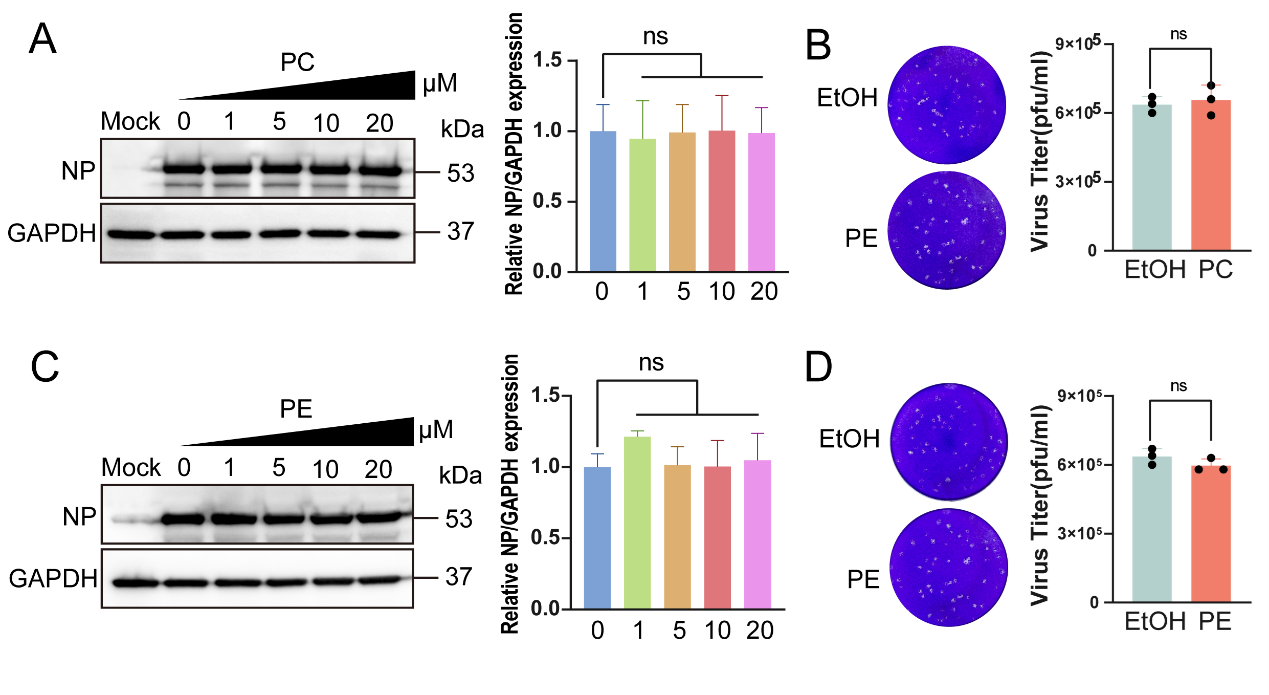

Supplement: Figure S2.tif [file KVIR_A_2580150_SM5717.tif]

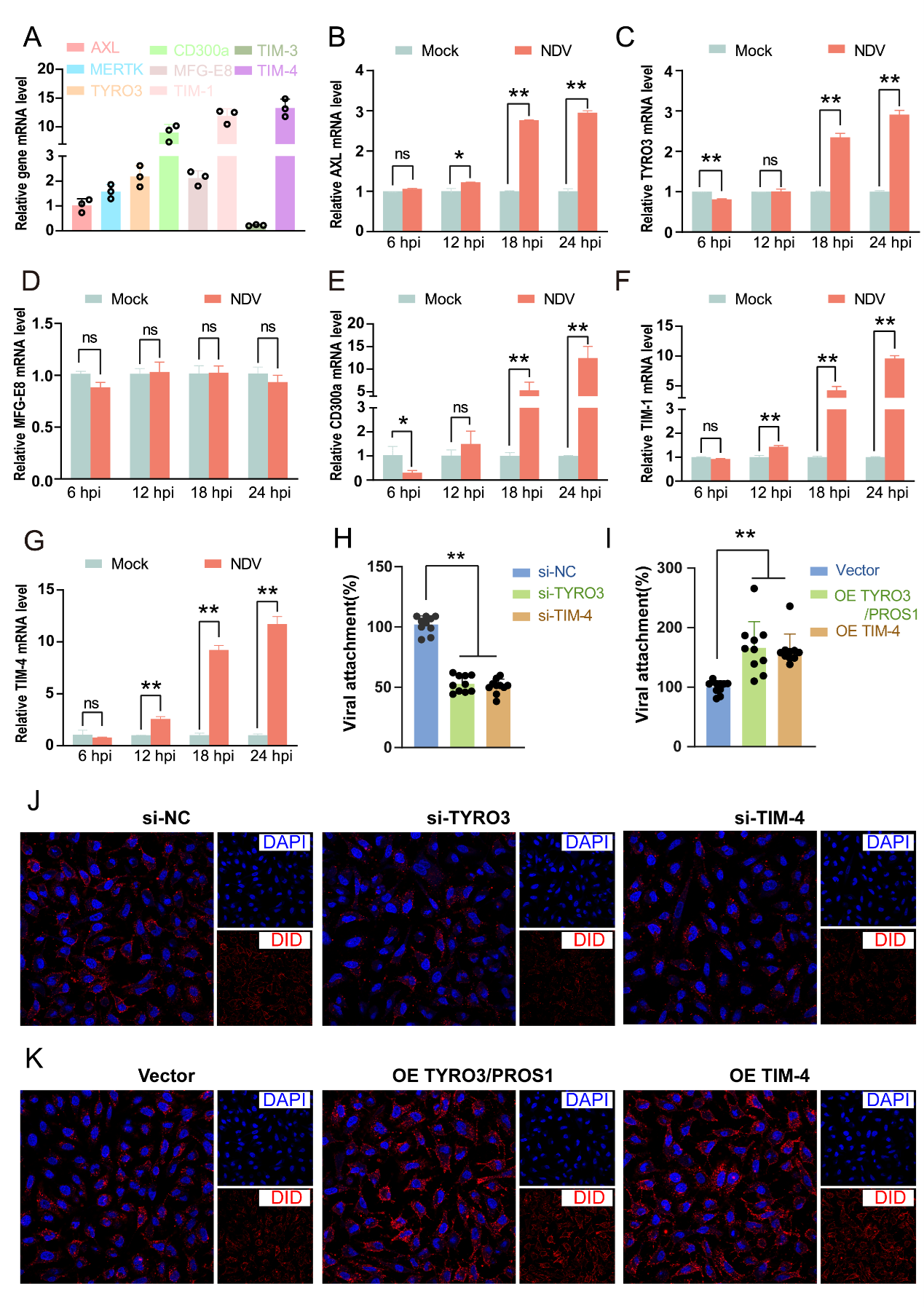

Supplement: Figure S6.tif [file KVIR_A_2580150_SM5716.tif]

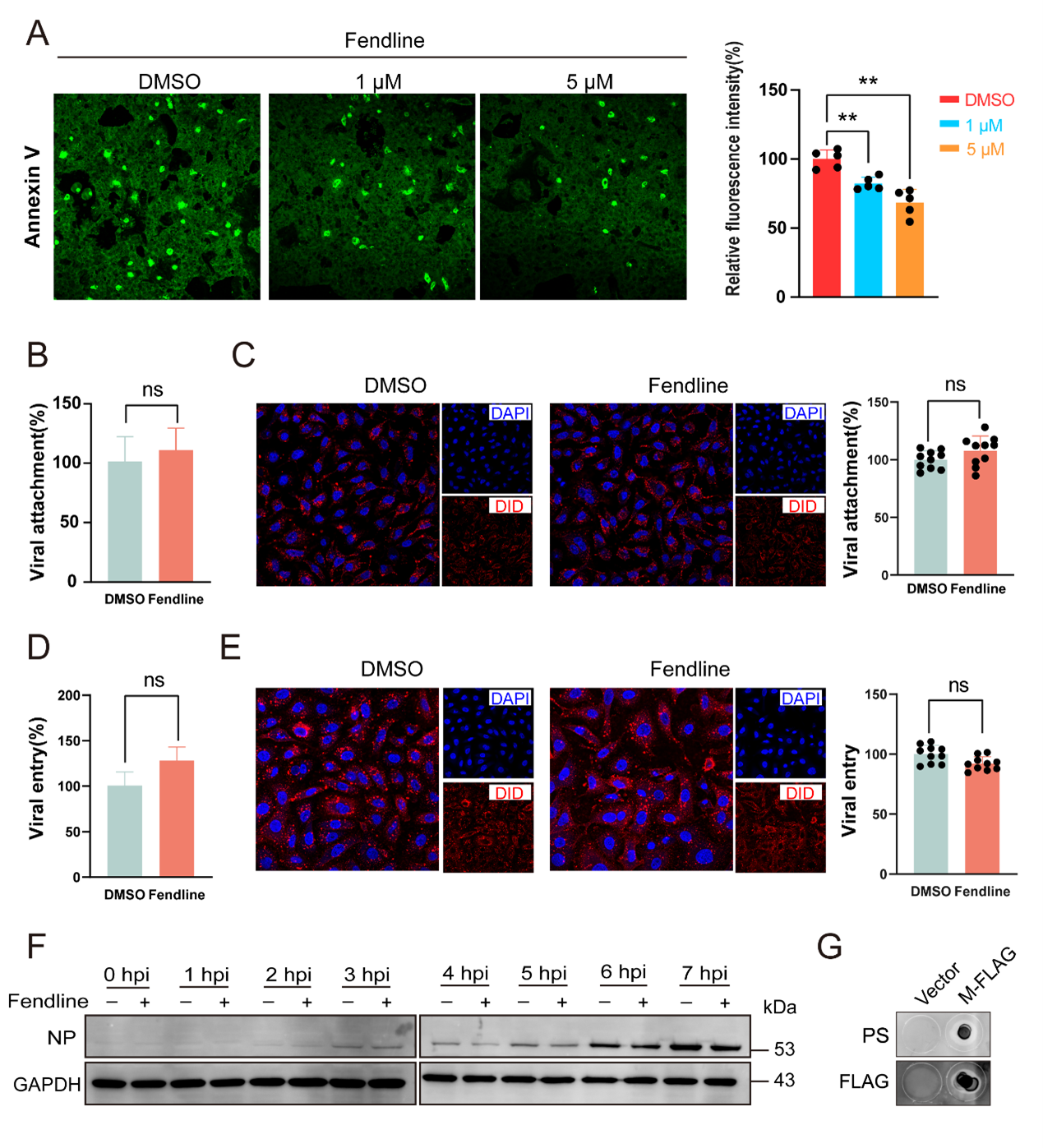

Supplement: Figure S1.tif [file KVIR_A_2580150_SM5715.tif]

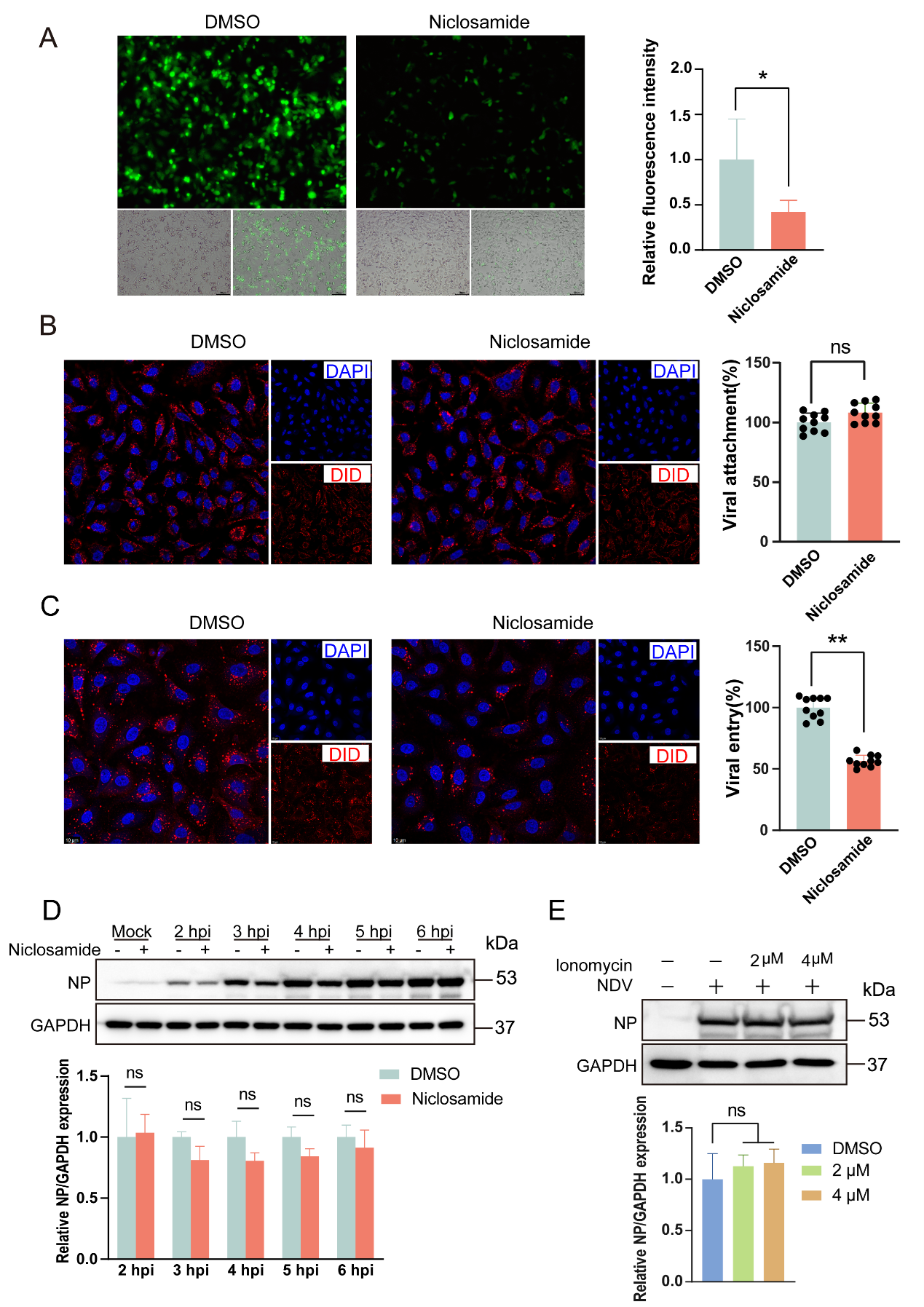

Supplement: Figure S5.tif [file KVIR_A_2580150_SM5714.tif]

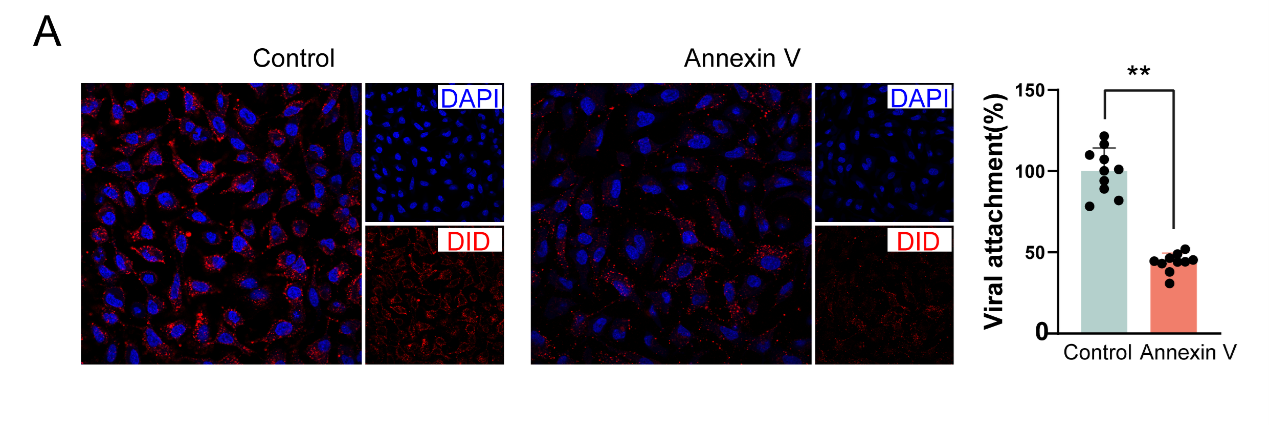

Supplement: Figure S3.tif [file KVIR_A_2580150_SM5713.tif]

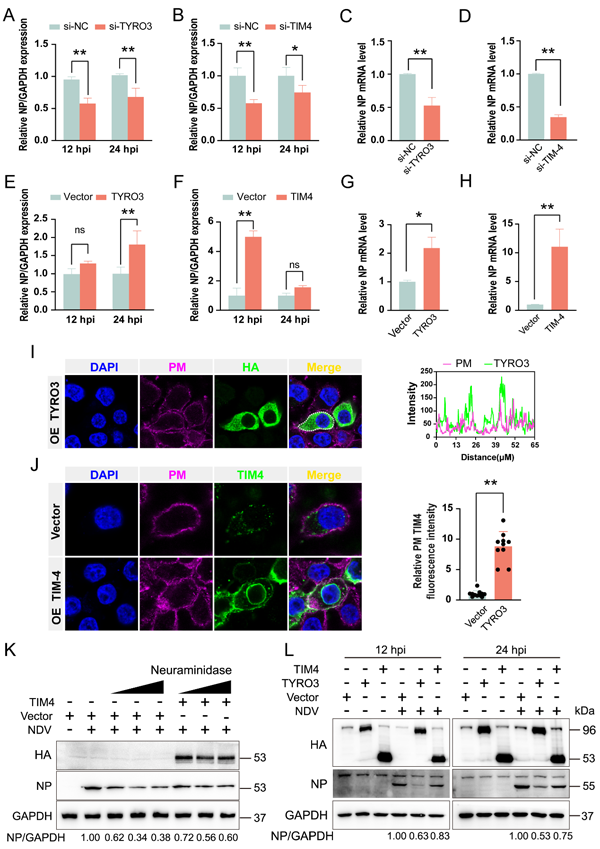

Supplement: Figure S7.tif [file KVIR_A_2580150_SM5712.tif]

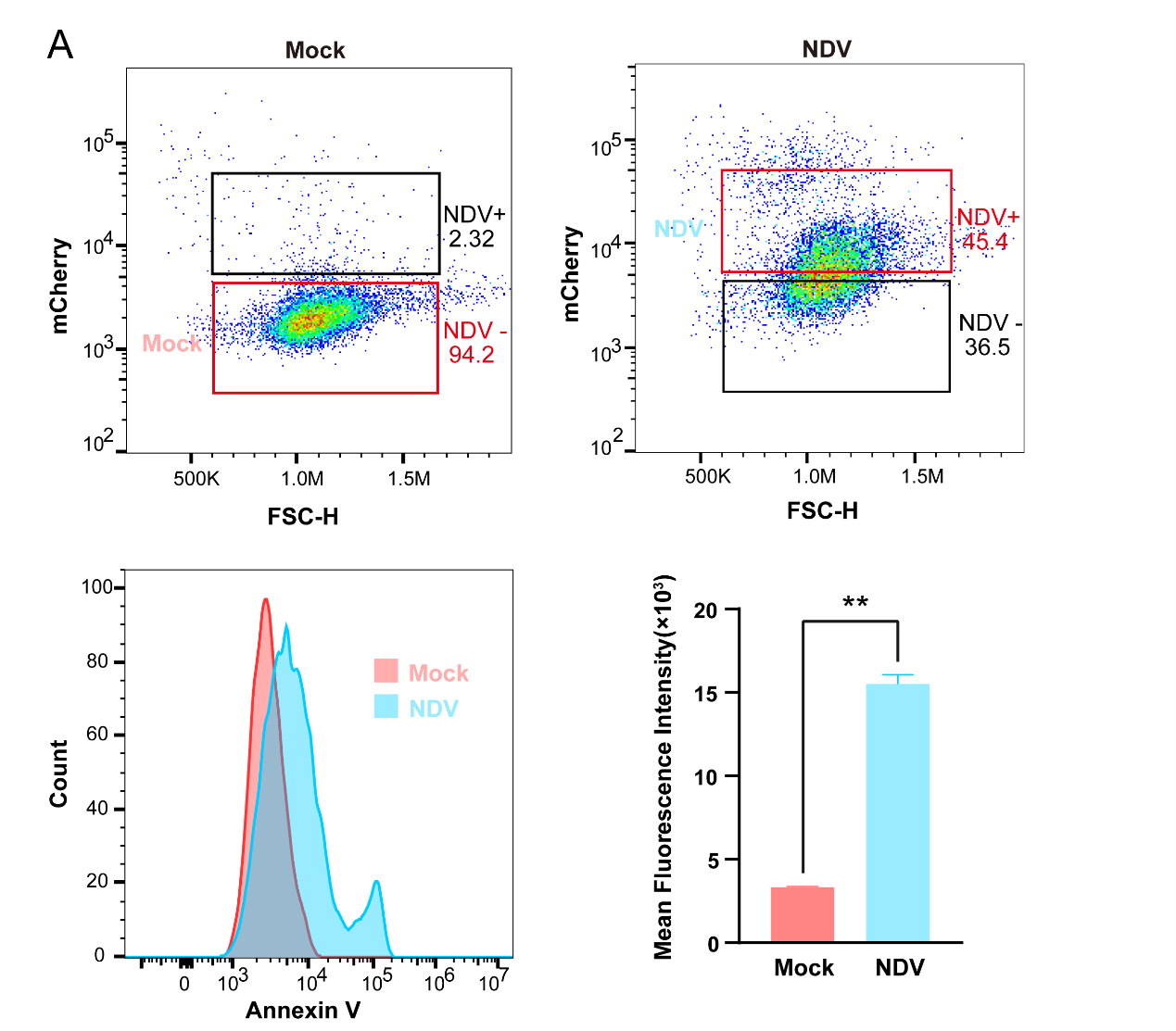

Supplement: Figure S4.tif [file KVIR_A_2580150_SM5711.tif]
