# Supplementary material for: Newcastle disease virus acquires phosphatidylserine through the budding process to enhance infectivity
Source: Virulence. 2025 Oct 28;16(1):2580150. doi: 10.1080/21505594.2025.2580150 (PMC12604639; doi:10.1080/21505594.2025.2580150)
Supplement: Supplementary Material 0902 final.docx [file KVIR_A_2580150_SM5710.docx]

**Supplementary Figures**

**Figure legend**

**Figure S1.** Related to Figure 1. (A) Total PS was visualised by fluorescence microscopy after Fendiline treatment and Annexin V labelling. (B and C) Cells were pre-treated with Fendiline or vehicle, exposed to NDV or DiD-NDV for 1 h at 4 °C, washed, and bound virions quantified by RT-Qpcr of cell-associated genomes (B) and confocal microscopy (mean DiD fluorescence,C). (D and E) After the same pre-treatment, cells were shifted to 37℃ for 1 h to permit internalisation, and internalised genomes were quantified by RT-qPCR(D) or imaged by confocal microscopy (E). (F) Cells were pre-treated with Fendiline or vehicle, Early replication kinetics (6 hpi) were monitored by Western blot for NP. (G) VLPs released from M-transfected HEK-293T cells were concentrated and analysed by dot-blot using anti-PS or anti-FLAG antibodies.

**Figure S2.** Related to Figure 2. (A-D) Parallel experiments were performed with exogenous phosphatidylcholine (PC) or phosphatidylethanolamine (PE) to exclude non-specific lipid effects; replication was monitored by Western blot and plaque assay.

**Figure S3.** Related to Figure 3. (A) NDV virions were pre-incubated with Annexin V to mask surface-exposed PS, then allowed to adsorb to A549 cells at 4 °C for 1 h. Virus binding was visualised by confocal microscopy using DiD-labelled NDV, and quantified as the mean fluorescence intensity of cell-associated virions (n = 10).

**Figure S4.** Related to Figure 4. (A) A549 cells were infected with NDV-mCherry (MOI = 5). At 12 hpi, mCherry fluorescence indicated infected cells, whereas surface-exposed PS was revealed by FITC–Annexin V staining.

**Figure S5** Related to Figure 7 (A) NDV-GFP replication in Niclosamide- or mock-treated cells was visualised by fluorescence microscopy. (B and C) DiD-NDV binding (B) and internalisation (C) were quantified by confocal microscopy after Niclosamide pre-treatment. (D) Cells were either pre-treated with Niclosamide or mock-treated, Early replication (6 hpi) was monitored by Western blot. (E) Ionomycin-treated (4μM) cells were infected and NP levels analysed by Western blot.

**Figure S6.** Related to Figure 8. (A) Basal mRNA levels of candidate PS receptors in A549 cells. (B-G) Time-course expression of these receptors after NDV infection. (H-K) Confocal quantification of DiD-NDV binding after TYRO3 or TIM-4 knock-down (H, J) or over-expression (I, K).

**Figure S7.** Related to Figure 8. (A and B) Densitometric quantification of NP protein from Western blots after siRNA-mediated knock-down of TYRO3 or TIM-4. (Cand D）Corresponding NP mRNA levels determined by RT-qPCR. (E) Confocal microscopy confirmed the plasma-membrane localization of ectopically expressed TYRO3 (F) Surface expression and membrane localization of over-expressed TIM-4 were similarly verified. (G and H) Densitometric quantification of NP protein following TYRO3/TIM-4 over-expression. (I and J) NP mRNA after TYRO3/TIM-4 over-expression. (K) TIM-4-over-expressing cells were pre-treated with increasing concentrations of neuraminidase for 1 h prior to NDV infection; NP protein was assessed by Western blot. (L) TYRO3/TIM-4 over-expression resulted in reduced AIV NP expression.
